# Exploring room-temperature transport of single-molecule magnet-based molecular spintronics devices using the magnetic tunnel junction as a device platform†

**DOI:** 10.1039/c9ra09003g

**Published:** 2020-03-31

**Authors:** Pawan Tyagi, Christopher Riso, Uzma Amir, Carlos Rojas-Dotti, Jose Martínez-Lillo

**Affiliations:** Department of Mechanical Engineering, University of the District of Columbia 4200 Connecticut Avenue NW Washington DC-20008 USA ptyagi@udc.edu; Instituto de Ciencia Molecular (ICMol), Universitat de València C/Catedrático José Beltrán 46980 Paterna València Spain f.jose.martinez@uv.es

## Abstract

A device architecture utilizing a single-molecule magnet (SMM) as a device element between two ferromagnetic electrodes may open vast opportunities to create novel molecular spintronics devices. Here, we report a method of connecting an SMM to the ferromagnetic electrodes. We utilized a nickel (Ni)–AlO_*x*_–Ni magnetic tunnel junction (MTJ) with the exposed side edges as a test bed. In the present work, we utilized an SMM with a hexanuclear [Mn_6_(μ_3_-O)_2_(H_2_N-sao)_6_(6-atha)_2_(EtOH)_6_] [H_2_N-saoH = salicylamidoxime, 6-atha = 6-acetylthiohexanoate] complex that is attached to alkane tethers terminated with thiols. These Mn-based molecules were electrochemically bonded between the two Ni electrodes of an exposed-edge tunnel junction, which was produced by the lift-off method. The SMM-treated MTJ exhibited current enhancement and transitory current suppression at room temperature. Monte Carlo simulation was utilized to understand the transport properties of our molecular spintronics device.

## Abstract 2

A device architecture utilizing a single-molecule magnet (SMM) as a device element between two ferromagnetic electrodes may open vast opportunities to create novel molecular spintronics devices.

## Graphical abstract

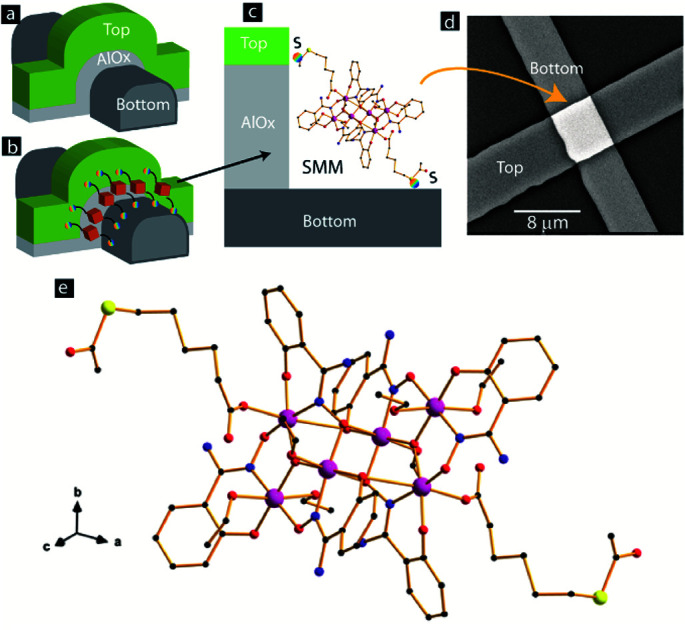

## Introduction

Single-molecule magnets (SMMs) are one of the most exciting class of molecules possessing tunable spin state for a wide range of applications and exhibit Berry phase-like quantum mechanical phenomena.^1^ SMMs are also highly promising for quantum computation applications.^2^ However, further advancement in producing SMM-based molecular devices will require an efficient and mass fabrication approach to connect metallic leads to this type of molecular system. To date, only planar nanogap junction-based devices, where a planar nanogap separates two gold electrodes, have been utilized.^2*c*,3^ The planar nanogap junction approach gives <10% yield and is primarily limited to gold metal serving as the source and drain electrode.^2*c*^ However, SMMs can behave very differently when connected to a variety of metallic electrodes. One major focus in the field of molecular spintronics is in the scope of simultaneously connecting an SMM to two ferromagnetic leads placed at the nanoscale gap. It will be intriguing to explore how spin-polarized transport from ferromagnetic electrodes can be used to manipulate and detect spin transport *via* an SMM with a tunable spin state. The impact of SMM interactions when strongly bonded to two ferromagnetic electrodes, not simply chemisorbed onto one ferromagnet, may modify the magnetic properties of the ferromagnetic film itself and hence produce spinterface-like devices for novel applications.^4^ The SMM interaction with ferromagnetic electrodes can nucleate local phenomena that may penetrate deep into the ferromagnetic electrodes, due to the presence of long-range magnetic ordering within a typical ferromagnet. To advance the possibilities mentioned above, we have attempted to test if magnetic tunnel junctions (MTJs) can be utilized as a test bed to study SMMs. An MTJ is basically a vertical nanogap junction where the gap between two ferromagnetic electrodes can be controlled to angstrom level *via* controllable thin-film deposition in sputtering machines that are widely available in small and large institutions. To study SMMs, we utilized exposed-edge MTJs produced by the lift-off methods established in our previous work.^5^ SMMs and insulator make parallel connections between two metal electrodes. This MTJ-based molecular spintronic device (MTJMSD) fabrication approach brings enormous advantages over conventional schemes and solves critical issues, such as oxidation of ferromagnetic electrodes.^6^ MTJMSDs have been successful in observing a number of intriguing and interesting phenomena by enabling magnetic molecule-induced strong exchange coupling between the ferromagnetic electrodes of an MTJ.^5,7^ Previously, we have utilized this MTJMSD approach to investigate organometallic molecules.^5^ Here, we report our first experimental results regarding the utilization of this MTJMSD approach to investigate the transport properties of SMMs at room temperature.

## Experimental details

**Fig. 1:**
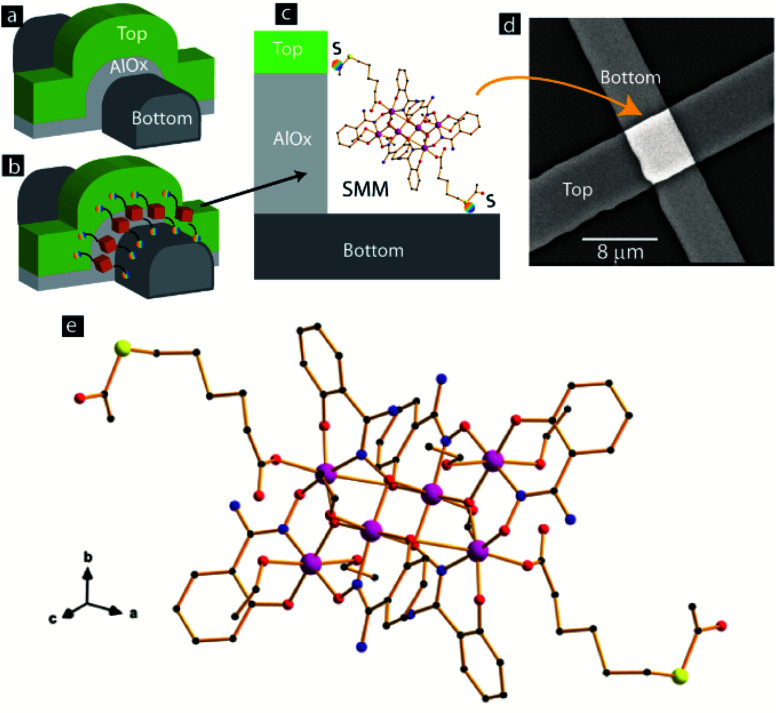
3D view of MTJ with exposed side edges (a) before and (b) after the bridging of SMM channels. (c) Magnified view of one SMM covalently bonded with two Ni electrodes. (d) SEM of a complete SMM-based MTJMSD. (e) View along the crystallographic [111] direction of the molecular structure of the SMM. H atoms and ethanol molecules of crystallization have been omitted for clarity. Color code: pink, Mn; yellow, S; red, O; blue, N; black, C.

In this study the MTJMSD mainly employed nickel (Ni) as the ferromagnetic electrodes. To identify the temperature limit up to which Ni could be heated without oxidation, a reflectance *vs.* temperature study was conducted (ESI, Fig. S1†). The MTJ test bed for studying the SMM-based molecular devices was fabricated by the lift-off method described in detail elsewhere,^8^ and also in the ESI for this paper (Fig. S2†). The protocol for this oxidation study is the same as that utilized in our previous work.^6^ This reflectance study suggested that the Ni film did not show any noticeable change in reflectance up to 90 °C (Fig. S1†). Hence, the temperature for the fabrication steps of the MTJMSD, where Ni was in the ambient state, was limited to 90 °C. For the MTJ test bed fabrication, we utilized tantalum (Ta) as a seed layer to promote adhesion between the oxidized silicon substrate and the bottom Ni electrode. We used alumina (AlO_*x*_) as the insulating spacer between the two ferromagnetic electrodes of different thickness. Our MTJ test bed with exposed side edges (Fig. 1a) possessed a configuration of Ta (5 nm)/Ni (20 nm)/AlO_*x*_ (2 nm)/Ni (10 nm). We kept the different top and bottom electrode thicknesses to produce a difference in magnetic coercivity, so as to acquire the ability to control the magnetization of thinner film at relatively low magnetic field compared with thicker Ni film.^9^ A three-dimensional (3D) perspective view of the exposed side of the MTJ is shown before (Fig. 1a) and after (Fig. 1b) hosting the molecules. Fig. 1c shows the connection of each SMM with the two metal electrodes, with the help of the thiol functional group. For SMM bridging, all the junctions were simultaneously submerged under the same SMM solution drop. For SMM bridging between Ni electrodes, we utilized the previously published electrochemical method.^8^ After molecular treatment, the excess SMM solution was washed off using ethanol. Subsequently, the sample was cleaned and dried before conducting microscopy and transport (*I*–*V*) studies.

In the MTJMSD, the SMMs and insulator make parallel connections between two metal electrodes. Based on the SMM size and available exposed lengths, we estimated that ∼10 000 SMMs could be connected between electrodes. The scanning electron microscopy (SEM) image in Fig. 1d shows the top view of an MTJMSD. The crystal structure of the SMM used in this paper has been reported elsewhere.^10^ Nevertheless, we discuss here certain structural features that are useful for understanding SMM characteristics and the possible effects on the studied molecular device. The magnified version of the SMM molecular structure is shown in Fig. 1e. This molecule crystallizes in the monoclinic system with space group *P*2_1_/*c*, and its crystal structure is made up of neutral hexanuclear [Mn_6_] complexes, along with ethanol molecules of crystallization. It has structural features in common with other Mn_6_ SMMs based on the salicylamidoxime ligand.^11^ Each hexanuclear [Mn_6_(μ_3_-O)_2_(H_2_N-sao)_6_(6-atha)_2_(EtOH)_6_] [H_2_N-saoH = salicylamidoxime, 6-atha = 6-acetylthiohexanoate] complex contains two symmetry-equivalent [Mn_3_(μ_3_-O)] triangular moieties, which are linked by two phenolate and two oximate O atoms. The six Mn^III^ ions exhibit distorted octahedral geometries with the Jahn–Teller axes approximately perpendicular to the [Mn_3_(μ_3_-O)] planes. The monodentate carboxylate ligand is coordinated on Mn(3) and on its symmetry equivalent. The remaining coordination sites on the Mn^III^ ions are occupied by ethanol molecules. The Mn–N–O–Mn torsion angles of the [Mn_3_(μ_3_-O)(H_2_N-sao)_3_] triangular unit are 38.9, 36.5 and 26.0°. The intramolecular S⋯S separation is *ca.* 23.0 Å^10^. We studied the MTJ test bed and SMM-treated MTJs with SEM and atomic force microscopy (AFM). We utilized a Phenom XL scanning electron microscope and a NaioFlex atomic force microscope for the microscopy study. The average width of the top and bottom electrodes was in the range 4–8 μm. Generally, the area of the MTJ junction was ∼40 μm^2^. Current–voltage (*I*–*V*) studies were performed on all the MTJs, before and after treating with the SMM or bridging SMM channels between two Ni electrodes. For the *I*–*V* studies, we utilized Keithley source meters (model 2420 and model 6430) connected to a biaxial cable and low-noise micromanipulator probes placed in a metal Faraday cage.

## Results and discussion

**Fig. 2:**
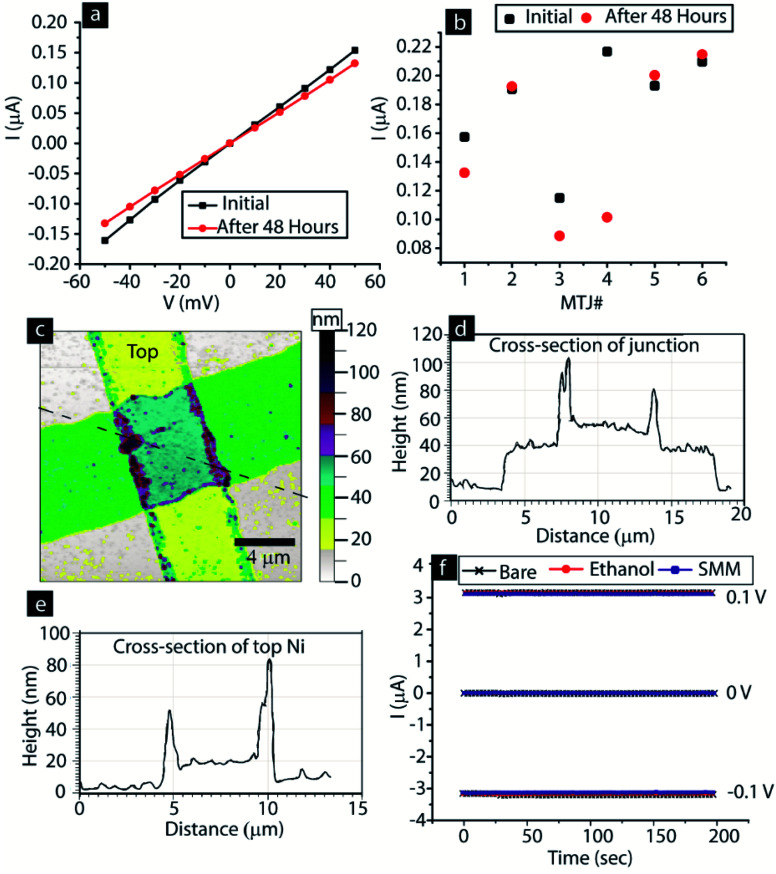
(a) Representative *I*–*V* of a bare MTJ after 48 hours. (b) Variation in current of six MTJs at 50 mV after 48 hours. (c) AFM image showing topography of an MTJ. (d) AFM measurements of the cross-section of the junction along the dashed line in panel (c). (e) AFM measurements of the cross-section of top Ni electrode. (f) Stability of the top Ni electrode state subjected to alternating ±0.1 V in the bare state, after immersion in ethanol, and after immersion in SMM solution in ethanol solvent.

We first focused on ensuring that the MTJ test beds were robust and utilized our well-established method for producing a high quality tunnel barrier.^6,7,12^ In the MTJMSD approach, instabilities in the MTJs are likely to arise due to (a) weak tunnel barrier that keeps degrading to the resistor like state, (b) high leakage current *via* spikes at the boarder of the photolithographically defined bottom electrode, and (c) potential chemical etching of the ferromagnetic electrodes under the effect of solvent and the SMM solution in ethanol. To produce a stable tunnel barrier for this study we utilized the previous optimized recipe for AlO_*x*_ deposition.^12*b*^ There are several insightful ways to study anomalies regarding MTJ tunnel barriers.^13^ According to our empirical understanding, an ∼2 nm tunnel barrier deposition is mainly impacted by the relaxing mechanical stresses.^12*b*^ We observed that tunnel barriers that are of high quality generally remain stable or slightly improve over a period of 48 hours (Fig. 2a and b). The mechanism behind the improvement in tunnel barrier quality is seemingly related to the relaxation of mechanical stresses in AlO_*x*_.^12*b*^ However, an in-depth analysis on this topic is beyond the scope this paper. In the present case, the MTJ test beds showed slight improvement (Fig. 2a). Data were taken from six representative MTJs that did not show any sign of degradation (Fig. 2b).

We also prevented transport leakage *via* the notches along the photolithographically defined bottom electrode side edges (Fig. 2c and d). According to our previous experience a cross-junction MTJ is highly unstable if side edges of the bottom electrode possess notches. These notches become a hot spot of charge transport, irrespective of the quality of the tunnel barrier in the planar area. Notches at the edge of photolithographically defined thin films were avoided by developing an undercut photoresist profile, as discussed in our previous work.^12*c*^ We produced all MTJ test beds with bottom electrodes possessing tapered side edges (Fig. 2c). The AFM cross-sectional image of the junction area shows that the bottom electrode was well rounded (Fig. 2d). We did not apply the improvised photolithography recipe of producing tapered edges for the top electrode, as top electrode edges do not interfere with the AlO_*x*_ tunnel barrier stability (Fig. 2e).

We also ensure that the MTJ test beds are fully intact after the interaction with the SMM solution in ethanol. The AFM study shown in Fig. 2c and d is on an MTJ that was treated with SMM solution in ethanol (SMM dissolved in ethanol). We found that all the nickel electrodes were fully intact and there was no sign of any chemical etching. This AFM study supports the SEM image of an MTJMSD shown in Fig. 1d (*i.e.* SEM image of SMM-treated MTJ test bed). The SEM and AFM studies confirmed that we did not cause any chemical etching of the Ni electrode. To triple-check that ethanol solvent, or SMM solution in ethanol, does not cause any damage (that could be seen in the AFM and SEM images) to the electrode, we conducted the transport study *via* the top electrode under different conditions (Fig. 2f). We chose the top electrode because this is nearly half of the thickness of the bottom electrode and will be able to respond readily to chemical etching. We alternated bias on the bottom electrode between 0.1 V and −0.1 V for 200 s (Fig. 2f). We could not see any difference in transport *via* the top nickel electrode due to prolonged exposure to ethanol and to SMM solution in ethanol. This experiment was repeated three times, each for 200 s, and no changes were observed. We also ensured that air exposure did not create any instability by oxidizing the Ni electrodes. We have already discussed that our MTJMSD fabrication approach is optimized by utilizing our discovery that most of the ferromagnets start oxidizing significantly after 90 °C (Fig. S1†).^6^ To further verify this, we also conducted *I*–*V* studies three years after device fabrication and found no change in the ∼10 nm thick Ni film.

In previous work, we and other groups have conducted additional control experiments to prove that molecular channels indeed serve as the effective conductance channel, compared with the tunnel barrier.^8,14^ Numerous previous studies have shown the ability of the tunnel junction-based molecular device to reverse the molecule effect on transport^8,14,15^ and, hence, unlike other approaches such as planar metal break-junctions based devices, it is far more suitable for making reliable molecular devices.

**Fig. 3:**
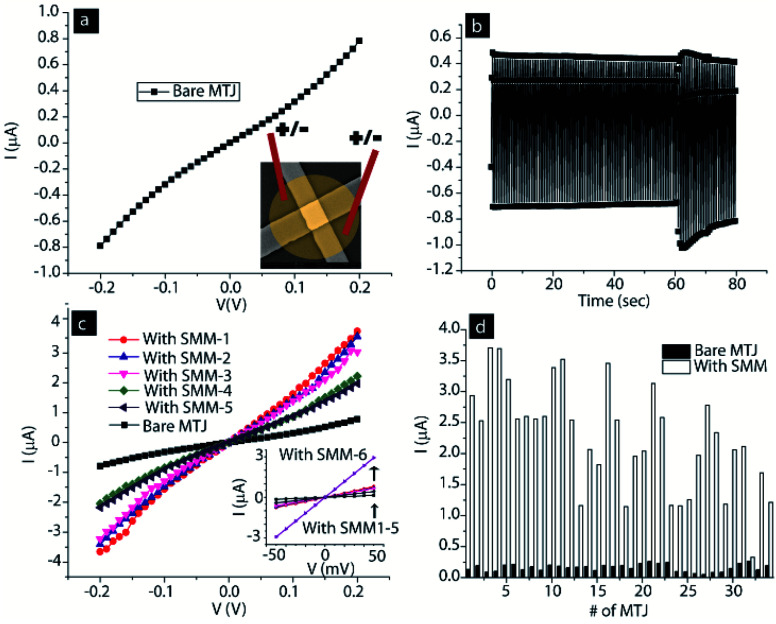
(a) Representative *I*–*V* of a bare MTJ. Inset shows that MTJs were submerged under SMM solution with two electrodes to apply ±100 mV. (b) Current *vs.* time spectra recorded between two electrodes placed in molecular solution on an MTJ. (c) Multiple *I*–*V* showing SMM effect on bare MTJ transport. (d) Histogram of 34 MTJs before and after hosting SMM channels along the edges.

An array of MTJs showing nonlinear *I*–*V* (Fig. 3a) relationships, a representative characteristic of tunneling-type transport, were subjected to the molecular bridging process. The inset image in Fig. 3a only shows the conceptual physical condition for one junction. The actual image of the immersion of all the junctions under the same molecular drop is shown in the ESI (Fig. S1†). For SMM bridging, all the 34 junctions were simultaneously submerged under the same molecular drop.

A typical current *vs.* time graph is shown in Fig. 3b. The *I*–*V* of the MTJ after interacting with the SMM showed a significant increase in the current of the MTJ (Fig. 3c). This increase in current indicates that the SMMs have successfully created additional transport channels across the AlO_*x*_ tunneling barrier, in agreement with the conceptual picture shown in Fig. 1c and our prior work in the area of MTJMSDs.^5,7*a*,8^ The central core of each SMM channel, as in the conceptual drawing in Fig. 1b and c, is connected to two metal leads *via* two hexane insulating tethers. The slowest step in transport *via* the SMM channel is expected to be the tunneling process *via* the hexane tethers. The length of each hexane tether is <1 nm, which is much smaller than the ∼2 nm AlO_*x*_ tunneling. Hexane tethers (<1 nm) are also free of structural defects compared with planar AlO_*x*_ tunneling barriers (∼2 nm). Two hexane tethers of each SMM make a strong Ni–S covalent bond with the Ni metal electrodes, resulting in a highly reproducible and well-defined interface (Fig. 1c). Hence, transport *via* SMMs is much more efficient compared with AlO_*x*_, and hence leads to a decrease in resistance of the MTJ test bed (Fig. 3c). Decrease in overall MTJMSD SMM- and MTJ-based molecular spintronics devices (SMM-MTJMSD) typically settled in the μA level current state. We also observed a similar phenomenon of reduction in resistance after bridging of another form of paramagnetic molecule between metal electrodes.^8^

We conducted multiple *I*–*V* studies right after SMM treatment to understand any initial dynamic process happening due to SMM and ferromagnetic electrode interactions. Six *I*–*V* studies on the freshly formed MTJMSD were different (Fig. 3c). The first three *I*–*V* studies were of a similar order of magnitude. However, the fourth and fifth *I*–*V* studies settled at a transient lower current state (Fig. 3c). Repeating the *I*–*V* measurements for a sixth time set it into the highest current state. This random switching between high, low and high current states after SMM bridging is believed to be due to the transient impact of SMM molecules on the ferromagnetic electrodes. SMMs are expected to establish highly efficient spin channels and a strong exchange coupling between the two ferromagnetic electrodes. In our prior study, we observed octa-metallic molecular cluster (OMC) paramagnetic molecules producing a transient effect that last from several minutes to hours. We are unable to explain the precise dynamics of ferromagnetic electrodes under the impact of molecular exchange coupling in the initial state. Based on our previous work,^5*a*,7*b*^ we believe that SMM-like paramagnetic molecules catalyze long-range changes on ferromagnets, which emanate from the molecule–ferromagnet interfaces. The SMM impacted regions might be propagating deeper into the ferromagnetic electrodes. During this period, a ferromagnetic electrode near the junction may experience competition between SMM-influenced regions and the original ferromagnetic electrode properties (*i.e.* before SMM interaction). We have previously observed the paramagnetic molecule impact spreading over regions of several micrometers.^5*a*,7*b*^ Further research may focus on investigation of the dynamic processes occurring on the freshly produced MTJMSDs.

**Fig. 4:**
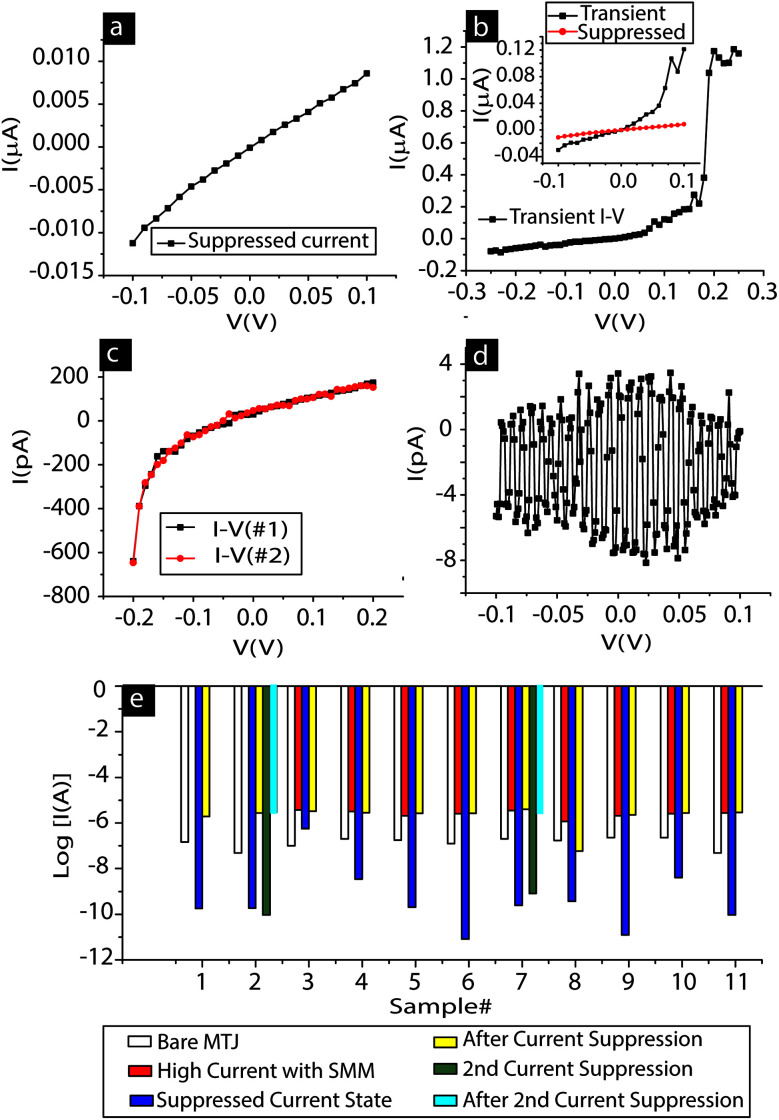
SMM-MTJMSD showing (a) suppressed current state and (b) nA level suppressed current state transitioning to the high μA level high current state. The suppressed current level could be quite stable (c) or in an ultra-low current state (d) where only noise could be recorded. (e) Histogram of 11 MTJs showing the suppressed current state after hosting SMM channels between Ni ferromagnetic electrodes.

The impact of the SMM was studied on 34 MTJs that were simultaneously treated with SMM solution to make molecular channels. All the 34 MTJs showed current enhancement (Fig. 4d). This study suggests that our MTJMSD fabrication process can have a nearly 100% device yield, which is mainly limited by the number of available MTJs per chip. In our previous work, we also demonstrated that several thousands of MTJ pillars, without any electrical connections, could be simultaneously transformed into molecular devices.^5*a*^ In the present case, the current for 34 MTJs at 50 mV increased from 0.16 ± 0.05 μA to 2.26 ± 0.86 μA (Fig. 4d).

It is noteworthy that our MTJs consist of Ni ferromagnetic electrodes and SMM paramagnetic molecules. The SMMs supposedly possess a net spin state, as expected with other SMMs.^1*b*^ Indeed, these Mn_6_ SMMs possess spin ground states varying from 4 to 12 at low temperature, depending on their flexible Mn–N–O–Mn torsion angles. In such cases, the spin state of the SMM has the possibility of interacting with the large magnetic ordering of the Ni electrodes *via* the <1 nm hexane tethers and Ni–S interfaces. It must be noted that the hexane molecule is an almost perfect tunneling channel with very high spin coherence length and time, due to significantly low spin scattering characteristics.^4*b*^ SMMs making covalent bond-based Ni–S interfaces on the side face of Ni ferromagnets are atomically similar for all the SMMs. SMM–Ni interfaces do not suffer from the interfacial roughness encountered in typical MTJ tunnel barriers. Hence, the SMM can become a strong bridge between two Ni ferromagnets. In this case, the SMM must be viewed as an extended Ni(bulk)-Ni(molecule affected)-Ni-S-hexane-[SMM Mn_6_ core]-hexane-S-Ni-Ni(molecule affected)-Ni(bulk) system. Our rationale for considering the SMM as an extended system is also based on our previous work with another form of MTJMSD involving OMC paramagnetic molecules and MTJs. In the previous work, the OMC was connected with two NiFe ferromagnetic films using ten-carbon-long alkane tethers and thiol bonds. In this case, the OMCs produced a robust antiferromagnetic coupling between the microscopic ferromagnetic electrodes. The OMC-induced exchange coupling was stable above 330 K and catalyzed the transformation of magnetic electrodes over areas of several micrometers.^5*a*,7*b*^ These OMC-based MTJMSDs provided direct evidence, in three independent magnetic measurements, that the paramagnetic molecules are no longer isolated from the electrodes.^5*b*,7*a*^ In the context of MTJMSDs, an OMC was operating far beyond its physical boundary. MFM-like room-temperature experimental studies showed that the OMC influence was observed as the extended system of NiFe(bulk)-NiFe(OMC impacted)-Ni-S-decane-[OMC]-decane-S-Ni-NiFe(OMC impacted)-NiFe(bulk) system. We also observed current suppression of several orders of magnitude on OMC-based MTJMSDs, which was only possible when ferromagnetic electrodes were impacted away from the physical locations of the OMCs.^5*a*,7*c*^ In the present case, SMM-MTJMSDs did not exhibit permanent current suppression. However, SMMs produced transient current suppression on MTJs.

A typical suppressed current state on SMM-treated MTJs is shown in Fig. 4a. Repeating *I*–*V* studies brought SMM-MTJMSDs into the high current state (Fig. 4b). The incubation period, when the SMM-MTJMSD was left idle for several hours to days, shifted many SMM-MTJMSDs from the ∼μA level high current state to a suppressed current state (Fig. 4a and c). In some instances, suppressed current states were rather robust and persisted for several hours, as observed during multiple *I*–*V* studies (Fig. 4c). Robust suppressed current states were observed from pA levels to almost complete current suppression, where only noise-like feature could be observed (Fig. 4d). The *I*–*V* for the SMM-MTJMSD shown in Fig. 4d resembles that of the MTJ with a ∼7 nm-thick tunnel barrier (ESI, Fig. S4†). Such, noise-like features appeared in multiple studies (ESI, Fig. S3 and S4†). The observation of current suppression was observed on 11 SMM-treated MTJs (Fig. 4e). Two MTJs, MTJ #1 and MTJ #2 (Fig. 4e) appeared in the suppressed current state right after the bridging of SMMs across the tunneling barrier. In all other cases, SMM-MTJMSD current increased at first and then settled in the temporary suppressed current state, and then again returned to the μA level high current state (Fig. 4e). Two samples, MTJ #2 and MTJ #7, showed current suppression twice. We studied the MTJMSDs for a period of four months. Every time we scanned the 34 junctions, we found 2–6 SMM-MTJMSDs in the suppressed current state, but the remaining SMM-MTJMSDs stayed in the high current state. It was apparent that the SMM-MTJMSD stable state is the high current state, as opposed to the stable suppressed current state observed in the previous study.^7*a*^ We carefully tested electrical leads and connections to ensure that the observed current suppression was only coming from the SMM-MTJMSD.

According to conventional MTJ-based spin-valve theory, antiparallel alignment of the magnetizations of the two ferromagnetic films produced the lowest current state.^16^ On the other hand, the parallel alignment of the ferromagnetic films produced the highest current state. In conventional spin-valve devices, an external magnetic field switches the direction of ferromagnetic electrodes between parallel and antiparallel states. According to traditional MTJ spin-valve theory, the difference between the MTJ high and low current state is mainly dependent on the spin polarization properties of the ferromagnetic electrodes. However, the spin polarization property is not the fundamental property of a ferromagnet. Spin polarization depends heavily on the medium present between the two ferromagnetic films. For example, the spin polarization of iron was drastically different when a MgO tunnel barrier replaced the AlO_*x*_ tunnel barrier.^16*b*,17^ An SMM-like paramagnetic molecule connected to two ferromagnetic electrodes *via* covalent bonding establishes strong exchange coupling with the two ferromagnetic electrodes, impacting the spin density of states.^5*a*^ If the exchange coupling is significantly strong, one can observe the effect on the microscopic junction area.^7*a*,7*c*^ In this paper, the observation of current suppression indicates that the SMM produced antiferromagnetic coupling between the two Ni electrodes. If the nature of molecular coupling is ferromagnetic, one could expect a permanent increase in device current. However, presumably, unlike in our previous work,^7*a*^ this SMM-induced antiferromagnetic coupling is unable to stabilize current suppression permanently. Also, the SMM core is paramagnetic, and when connected to two ferromagnetic electrodes it can influence what type of spin will cross over easily. This phenomenon is called spin filtering and can modify the Ni spin polarization. Our hypothesis that the SMM produces spin polarization and antiferromagnetic coupling leading to current suppression is also in agreement with our previous work on a very similar MTJMSD system.^5*a*,7*a*^ To further explain the various possibilities of SMM-induced exchange coupling between ferromagnetic electrodes, we discuss Monte Carlo simulations later in this paper.

**Fig. 5:**
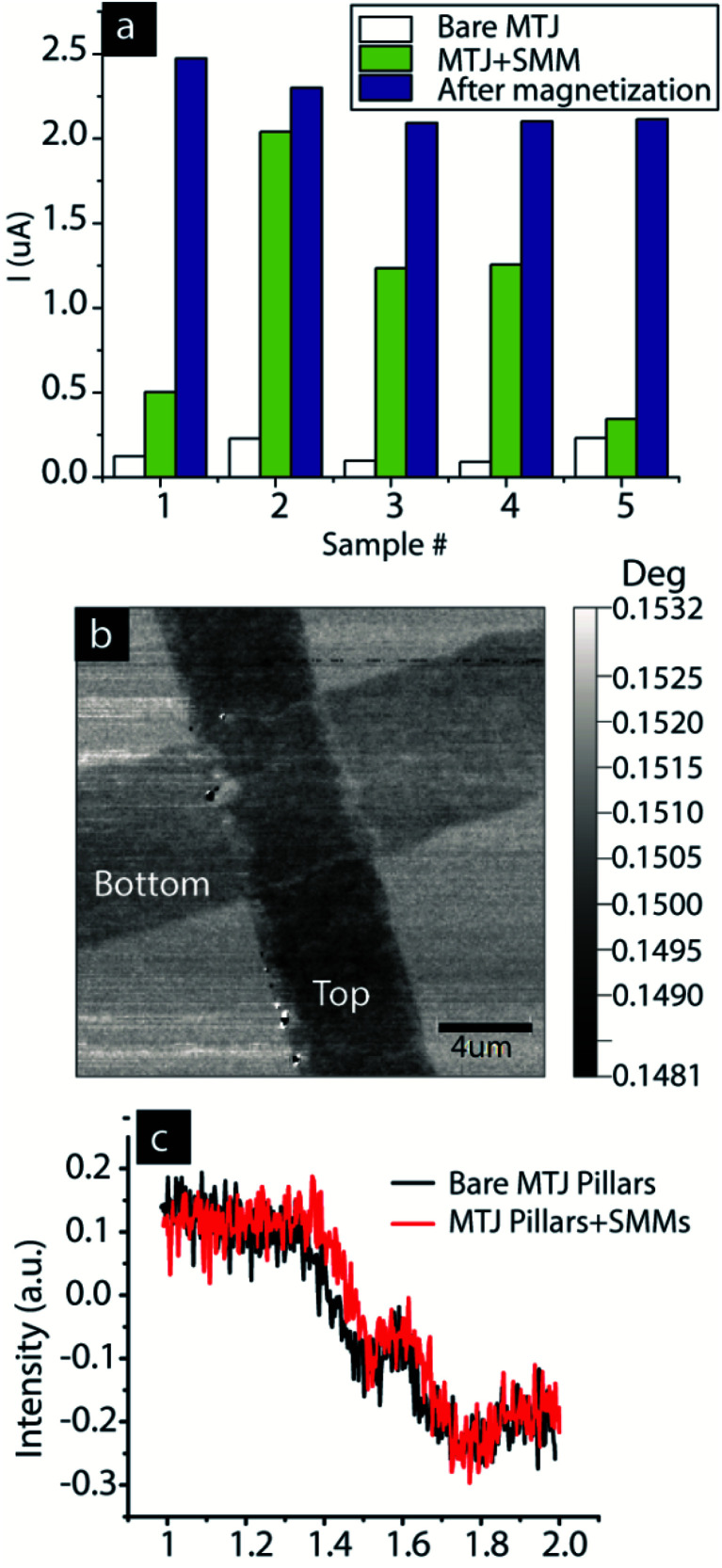
(a) SMM-MTJMSD showing magnetization's effect. (b) MFM of bare MTJ (c) FMR of an array of MTJ pillars before and after treating them with SMM.

We hypothesize that if SMM-MTJMSD transport is affected by the induced antiferromagnetic exchange coupling of the SMM with the magnetic electrodes, then the application of the magnetic field should produce a noticable effect. Next, we investigated the SMM-MTJMSD under magnetic field applied during the electrical measurement. Subjecting SMM-MTJMSD up to ∼500 Oe did not yield any noticeable change in magnetic transport (ESI, Fig. S5†). However, magnetizing the SMM-MTJMSD under ∼0.2 T magnetic field with the help of a permanent magnet promoted higher current state (Fig. 5a). We noted the currents of the five MTJs at 50 mV increased from 0.16 ± 0.07 μA to 1.08 ± 0.68 μA after hosting SMM channels (Fig. 5a). Magnetizing in the permanent magnetic field further increased the SMM-MTJMSD current to 2.26 ± 0.17 μA (Fig. 5a). We also attempted to measure the impact of SMM by carrying out magnetic measurements. We performed magnetic force microscopy (MFM) using a NaioFlex AFM. To prevent topographic effects from arising in the MFM, we kept 100 nm separation between the MTJMSD features and the AFM cantilever. We noted that before interacting with SMMs, a bare MTJ showed moderate magnetic contrast in the MFM scan (Fig. 5b). However, it was extremely challenging to get any conclusive MFM image and notice a substantial change in MFM contrast due to SMMs. We also employed ferromagnetic resonance (FMR) to study the SMM impact on an array of ∼20 000 MTJ cylindrical pillars. Sample preparation for the MTJ pillars was in accordance with our previously reported lift-off-based method.^5*a*^ FMR study was performed with NanoOsc Phase FMR at 10 GHz microwave frequency. FMR showed two overlapping resonance for the MTJ (Fig. 5c). FMR signal did not change noticeably after SMM interaction with the MTJ pillars. We surmise that either SMM was unable to impact the large enough population of 20 000 MTJ to produce detectable FMR signal, or SMM coupling between the two electrodes was not strong enough to provide a stable and noticeable change in the FMR signal. MFM and FMR were done at room temperature. In the future study, we plan to do low-temperature magnetic studies to understand the SMM effects on ferromagnetic electrodes.

To understand the role of the SMM in transforming the MTJ, we conducted Monte Carlo simulations (MCS). Generic MCS details about our approach are published elsewhere.^5*a*^ We represented the SMM-MTJMSD with a 7 × 10 × 10 Ising model, and a not-to-scale schematic is shown in ESI Fig. S6.† Each ferromagnetic electrode was attributed to a model dimension of 3 × 10 × 10 containing 300 atoms. A rim of molecules of dimension 10 × 10 was sandwiched between two ferromagnets (ESI Fig. S6†). In our previous work, we utilized such a model to provide insights and an explanation for the experimentally observed molecule-induced strong exchange coupling effect.^5*a*^ In the present case, the interaction of molecules placed along the central plane just along the edges was parametrically defined by the exchange coupling parameters. A unit vector was used to represent the spin of each ferromagnetic electrode atom and molecule. The initial state of the model was that all the spin vectors were aligned in the same direction. The molecule interactions with the top and bottom ferromagnetic layers were termed as *J*_SMM-T_ and *J*_SMM-B_, respectively. The energetics of reaching an equilibrium magnetic state of the MTJMSD can be defined by eqn (1).1
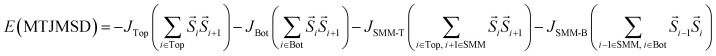


**Fig. 6:**
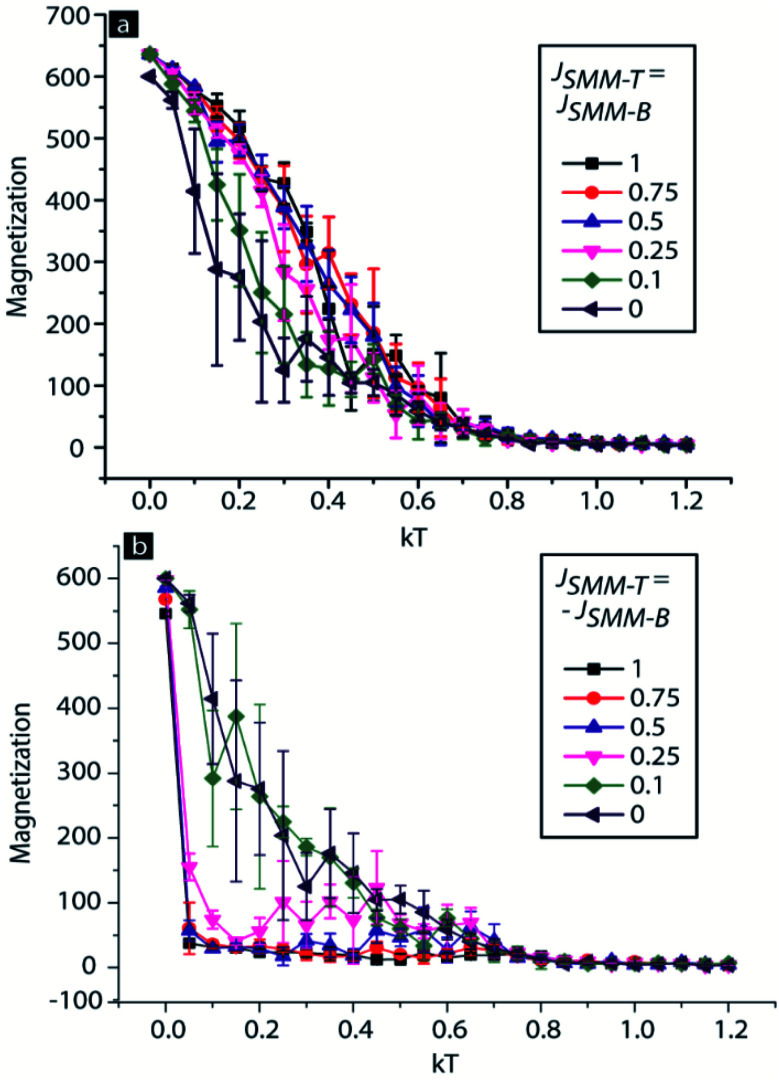
Magnetization *versus* thermal energy (*kT*) graph for the 3D Ising model of the SMM-based MTJMSD when *J*_SMM-T_ and *J*_SMM-B_ are of same magnitude and with (a) the same sign and (b) the opposite sign.

In eqn (1) atoms of the ferromagnetic electrodes and molecules are represented by the spin vector *S*. In the expression for *E*(MTJMSD) the *J*_Top_ and *J*_Bot_ are the Heisenberg exchange coupling strengths for the top and bottom ferromagnetic electrodes, respectively. The role of *J*_Top_ and *J*_Bot_ is critical in the MTJMSD. These two parameters are the sole reason for propagating the effect of the induced exchange coupling of the SMM from the tunnel junction edges to interior parts of the Ni electrodes. Each SMM molecule simultaneously connected top and bottom ferromagnetic electrodes, as in the schematic shown in Fig. 1b and the atomistic model discussed in the ESI (Fig. S6†). We varied the sign and magnitude of these *J*_SMM-T_ and *J*_SMM-B_ parameters and measured the magnetization ground state of the MTJMSD. The positive and negative sign of the exchange coupling parameters represented ferromagnetic and antiferromagnetic coupling, respectively. In the initial state, all the spin vectors were aligned in the same direction.^5*a*^ When *J*_SMM-T_ and *J*_SMM-B_ were 0, two ferromagnets were uncoupled. As the thermal energy (*kT*) increased magnetization kept decreasing, and at around *kT* = 1, the Curie temperature, the MTJMSD magnetization became zero (Fig. 6a). For the cases when both *J*_SMM-T_ and *J*_SMM-B_ were positive, the MTJMSD magnetization increased at a given *kT* with increasing coupling strengths, compared to the case where *J*_SMM-B_ = *J*_SMM-T_ = 0 (Fig. 6a). We studied *J*_SMM-T_ = *J*_SMM-B_ = 0.1, 0.25, 0.5, 0.75, and 1 to observe any potential transition. Increasing coupling strengths decreased spin fluctuations. However, increasing magnetization cannot explain the current suppression. According to well-established spin-valve theory^16*a*,18^ and the work of Petrov and co-workers,^19^ magnetic leads have to be antiparallel to each other to produce the least current state on an MTJ.

We calculated the magnetization for the case when *J*_SMM-T_ was of the opposite sign with respect to *J*_SMM-B_ (Fig. 6b). The magnitude of both parameters was equal and was the same as for Fig. 6b. When the magnitude of *J*_SMM-T_ and *J*_SMM-B_ was less than 0.25, the magnetization of the MTJMSD showed negligible change with respect to the case when two magnetic layers were uncoupled, *J*_SMM-T_ = *J*_SMM-B_ = 0. However, for the weak coupling, the magnetization of the MTJMSD was significantly noisy, indicating that two ferromagnets were switching between various magnetic alignments at fixed *kT*. As the molecular coupling strength was 0.25, the magnetization of the MTJMSD started settling at near-zero magnetization because two ferromagnetic electrodes are preferably aligned in opposite directions. However, 0.25 is still not strong enough to make two ferromagnets align perfectly antiparallel at different *kT*. In this state, two electrodes cancel the magnetization of each other.^18^ This molecule-induced antiparallel state is also responsible for the suppressed current state.^5*b*,7*a*^ However, if the molecular coupling strength is in between 0.1 and 0.25, overall the MTJMSD may be in an unstable state (Fig. 6b). At a given thermal energy, an MTJMSD may switch back and forth in the low and high current state, as in the phenomenon observed in this paper. If the magnitude of the molecular coupling strength increases beyond 0.25, very stable antiparallel alignment of ferromagnetic layers will be achieved, and will be observed by means of the decreased magnetization.^5*a*^ In such cases, an MTJMSD will exhibit current suppression.^5*b*,7*a*^ In the previous study, with a different type of paramagnetic molecule, strong antiferromagnetic exchange coupling was produced between two ferromagnetic electrodes, leading to room-temperature current suppression and long-range impact on the magnetic properties of the ferromagnetic electrodes^5*a*,7*a*^.

One may argue that the Ni ferromagnets used in this study are not 100% spin polarized. In the generic MCS discussed here, we did not account for this fact about Ni. However, a large number of studies have demonstrated that the degree of spin polarization of a ferromagnetic electrode is a strong function of the inter-ferromagnetic electrode coupling.^9,20^ In the present case, we surmise that the SMM serves the role of a spin-filtering agent, impacting the spin polarization of the Ni. An SMM also strongly couples the Ni electrodes, to yield strong exchange coupling, which governs the alignment of the spin-polarized Ni electrodes. However, at higher thermal energy, the molecular coupling may make the Ni electrode alignment switch between parallel and antiparallel states, like the one seen in Fig. 6b. The present SMM-MTJMSD appears to be more stable in the higher current state compared to the suppressed current state.

## Conclusion

We demonstrated the use of the MTJ test bed-based approach for studying molecular systems with SMM behavior. Transport studies (*I*–*V*) were performed at room temperature and showed that the SMM generally increased the current of the host MTJs. Several MTJs also showed a temporary current suppression phenomenon. Magnetizing the SMM-based MTJMSD stabilized the high current state. This study showed that device yield could approach 100%, and mainly depended on the quality and availability of the MTJs (MTJs per chip). We also formed MTJMSDs in a way that does not lead to oxidation of the nickel ferromagnet. It is noteworthy that oxidation of the ferromagnet is cited as a major obstacle in fabricating molecular devices. SMM-based MTJMSDs produced a transient current suppression of several orders of magnitude. Future studies employing MTJs with various types of ferromagnetic electrodes and other varieties of SMMs will provide new insights. In future, we plan to pursue low-temperature transport studies under varying magnetic field and light irradiation to explore the impact of the quantum state of the SMMs on transport and to realize magnetoresistance-like switching mechanisms. In addition, we plan to conduct magnetometry on MTJMSDs with different ferromagnetic electrode compositions to create differences in magnetic anisotropy and saturation fields. Such variations in the ferromagnetic electrodes are expected to enable SMMs to have different effects on the MTJMSDs.

## Conflicts of interest

There are no conflicts to declare.

## Supplementary Material

RA-010-C9RA09003G-s001
