# Supplementary material for: Exploring room-temperature transport of single-molecule magnet-based molecular spintronics devices using the magnetic tunnel junction as a device platform
Source: RSC Adv. 2020 Mar 31;10(22):13006–15. doi: 10.1039/c9ra09003g (PMC9051408; doi:10.1039/c9ra09003g)
Supplement: RA-010-C9RA09003G-s001 [file RA-010-C9RA09003G-s001.pdf]

Electronic Supplementary Material (ESI) for RSC Advances.
This journal is © The Royal Society of Chemistry 2020

Supplementary Material

Exploring Single-Molecule Magnets (SMM) based Molecular Spintronics Devices by Using the Magnetic Tunnel Junction (MTJ) with Exposed Side Edges

Pawan Tyagi[1*], Christopher Riso[1], Amir Uzma[1], Carlos Rojas-Dotti,[2] Jose Martínez-Lillo[2*]

University of the District of Columbia, Department of Mechanical Engineering, 4200 Connecticut Avenue NW Washington DC-20008, USA[1]; Instituto de Ciencia Molecular (ICMol), Universitat de València, c/ Catedrático José Beltrán , 46980 Paterna, València, Spain[2]
Corresponding Author Email: ptyagi@udc.edu and F.Jose.Martinez@uv.es*

Nickel stability with temperature

MTJ testbed for studying SMM based molecular devices were fabricated by the lift off method that has been described in Fig. S2. In this study MTJMSD mainly employed nickel (Ni) as the ferromagnetic electrodes. To identify the temperature, limit up to which Ni could be heated without oxidation, a reflectance vs. temperature study was conducted (Fig.S1)

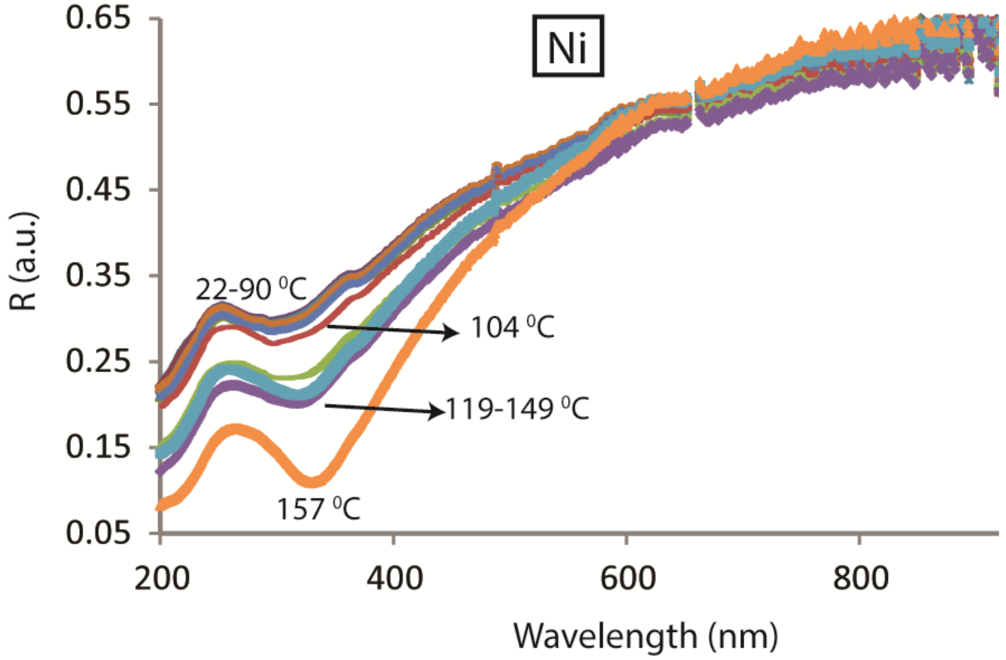

Fig. S1. Reflectance versus wavelength study of nickel film heated from RT to 157°C.

SMM based MTJMSD Fabrication:

For MTJ testbed fabrication a bottom electrode, comprising ~5 nm tantalum (Ta) seed layer and 20 nm Ni, was sputter deposited on the oxidized Silicon wafer (Fig. 2a). Subsequently, photolithography was performed to create a rectangular cavity pattern perpendicular to the bottom electrode direction (Fig. 2b). For photolithography, Shipley 1813 positive photoresist was spin coated at 4000 rpm and baked on a hot plate set at ~90 °C. It is noteworthy that during baking period bottom Ni is also covered by the ~2 µm photoresist that create a barrier between air and Ni during 1 min long baking. In the lithographically produced cavity, ~ 2 nm aluminum oxide (AlOx) (Fig. 1c) and ~ 10 nm Ni top metal electrode was deposited (Fig. 2d). In this study, we sputter deposited films using AJA ATC Orion 5UHV sputtering machine. Ni deposition was performed using RF sputtering target at 125 W gun power and 2 mTorr Ar pressure. AlOx was also deposited in the same sputtering machine, we utilized a previously published two-step AlOx growth method[1]. Lift-off of the photoresist removed excess AlOx and Ni top metal layer leaving a tunnel junction with exposed side edges (Fig. 2e). A 3D perspective view of the exposed side of the MTJ before (Fig. 2f) and after (Fig. 2g) hosting the single molecular magnets (SMM) molecules. Figure 1h shows the connection of each SMM with the two metal electrodes with the help of the thiol functional group. SMM was dissolved in ethanol to make ~ 1 mM solution and utilized electrochemistry to covalently connect SMM to the ferromagnetic electrodes. We have extensively discussed the electrochemical molecular bridging process in the prior work[2]. Tour group has produced excellent background research about the efficacy of electrochemistry based self-assembly process as compared to time-dependent self-assembly. SMM-MTJMSD's SEM image produced a top profile showing no sign of physical damage (Fig. S2i). All the MTJs were treated with SMM simulatenously by submerging under the same SMM soluton drop (Fig.S2j). After treatment for couple of minutes solution drop was removed and simples were dried before conducting transport studies. Based on SMM size and available exposed lengths, we estimated that ~10,000 SMM could be connected between electrodes.

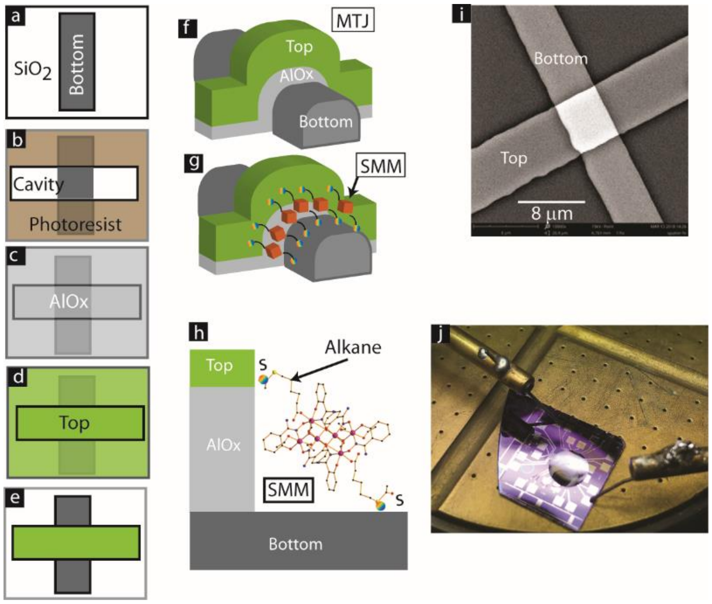

Fig. S2: Fabrication of MTJ with exposed side edges by (a) depositing bottom Ni electrode, (b) creating a photolithographically defined cavity for (c) depositing ~2 nm AlOx, and (d) top Ni electrode. (e) Liftoff produced an array of (e) MTJ cross junction with the (f) exposed sides along the top Ni electrode. (g) SMM molecules are bridged across AlOx insulator. (h) Each SMM possess alkane tethers to provide the connection with SMM core and making the covalent bond with Ni electrodes. (i) SMM-MTJMSD's SEM image produced a top profile showing no sign of physical damage. (j) All the MTJs were treated with SMM simulatenously by submerging under the same SMM soluton drop. After treatment for couple of minutes solution drop was removed and simples were dried before conducting transport studies.

Structure description of SMM (**1**).

The crystal structure of compound **1** has been reported elsewhere [3][1]. Nevertheless, we discuss here certain structural features that are useful to understand its behavior and possible effects on the studied molecular device. **1** crystallize in the monoclinic system with space group $P2_1/c$, and its crystal structure is made up of neutral hexanuclear Mn_6 complexes along with ethanol molecules of crystallization (Figure 1). It has structural features in common with others Mn_6 single-molecule magnets based on the salicylamidoxime ligand [4-10][2-9]. Each hexanuclear $[Mn_6(\mu_3\text{-}O)_2(H_2N\text{-}sao)_6(6\text{-}atha)_2(EtOH)_6]$ [H_2N-saoH = salicylamidoxime, 6-atha = 6-acetylthiohexanoate] complex contains two symmetry equivalent $\{Mn_3(\mu_3\text{-}O)\}$ triangular moieties, which are linked by two phenolate and two oximate O-atoms. The six Mn^{III} ions exhibit distorted octahedral geometries with the Jahn-Teller axes approximately perpendicular to the $\{Mn_3(\mu_3\text{-}O)\}$ planes. The monodentate carboxylate ligand is coordinate on Mn(3) and on its symmetry equivalent. The remaining coordination sites on the Mn^{III} ions are occupied by ethanol molecules. The Mn-N-O-Mn torsion angles of the $[Mn_3(\mu_3\text{-}O)(H_2N\text{-}sao)_3]$ triangular unit are 38.9, 36.5 and 26.0°. The intramolecular S⋯S separation is ca. 23.0 Å, whereas the shortest intermolecular S⋯S distance is 7.99 (2) Å [1].

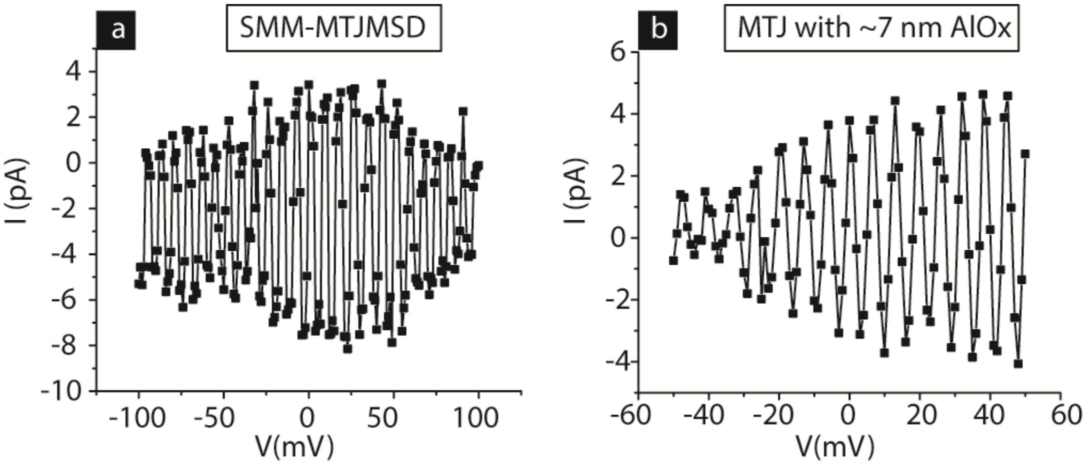

Figure-S3: Oscillatory I-V response at room temperature from (a) SMM-MTJMSD and (b) bare MTJ with 7 nm AlOx.

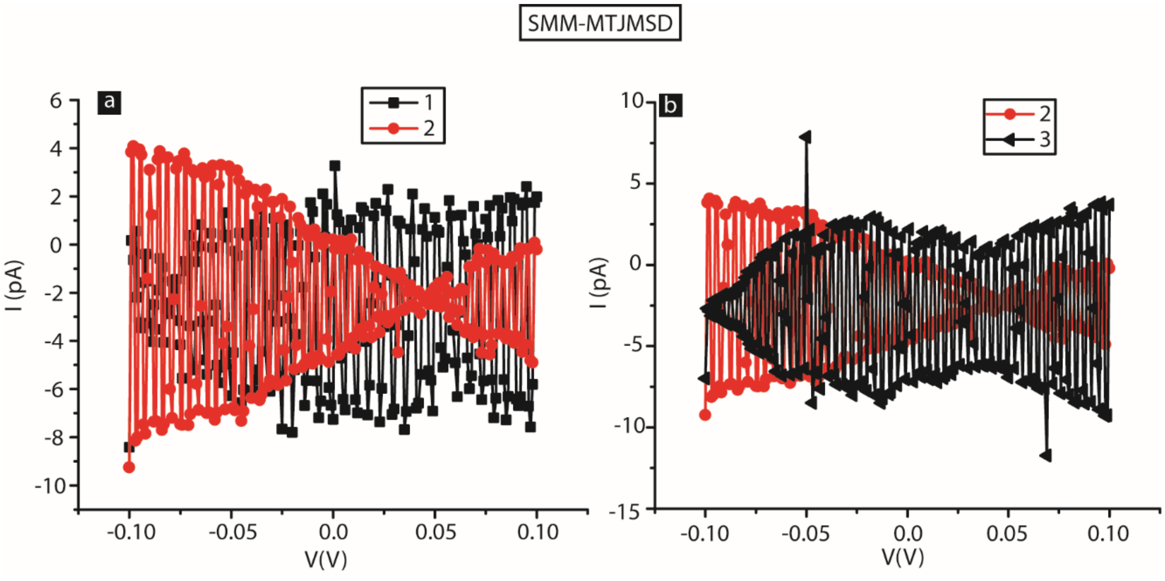

Figure-S4: Oscillatory I-V response at room temperature from a SMM-MTJMSD. (a) I-V #1 and I-V#2 were performed after 5 min Interval and (b) I-V #2 and I-V#3 were performed after 5 min Interval

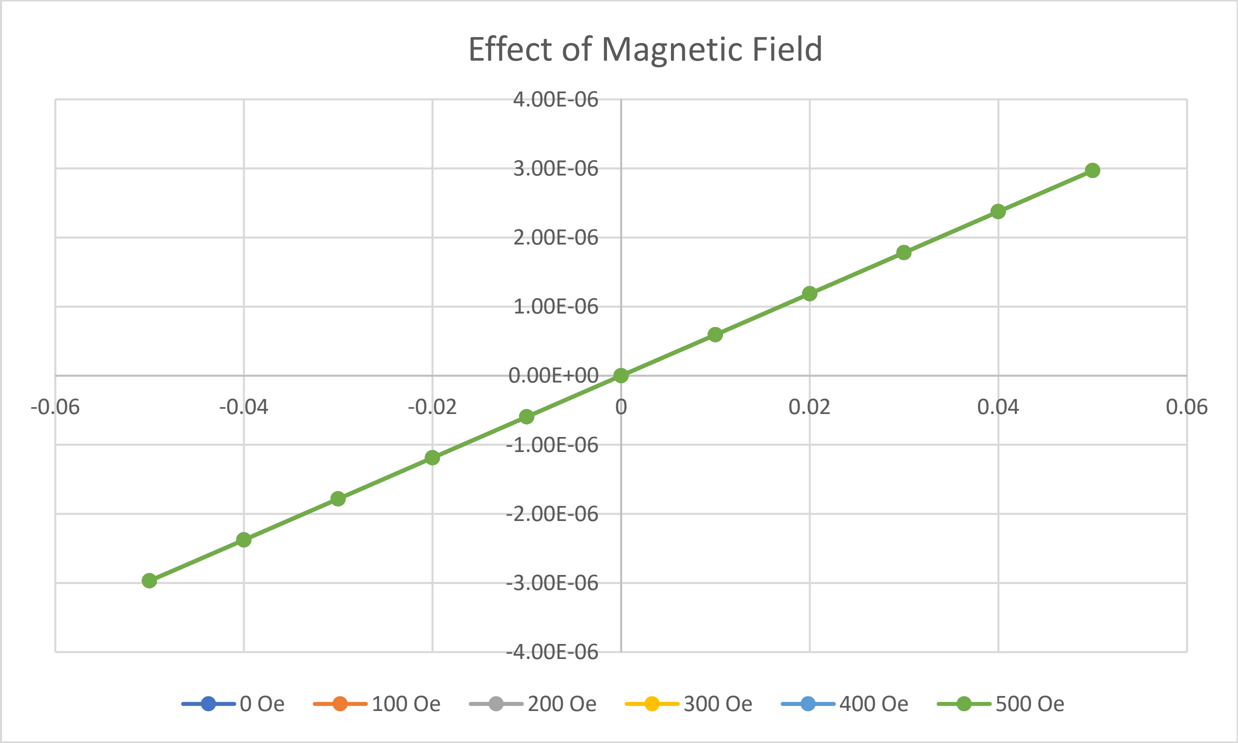

Figure S5: Effect of varying magnetic field from electromagnet on SMM-MTJMSD. Data points at individual fields are overlapping.

We also analyzed the transport data before and after transforming MTJ into the molecular device using the Brinkman tunneling model [11]. For this analysis, experimentally obtained I-V data was transformed into current density-voltage data and was fitted with a third-order polynomial to utilize the Brinkman model. The coefficients of the 3rd order polynomial are the coefficients of the analogous current vs. voltage graph one can obtain from the Brinkmann conductance equations mentioned below. For the samples on which I-V was performed for a shorter voltage range due to stability reason, we extrapolated the data in line with our previously published work[12]. Here we show the representative I-V curves that were fitted with the 3rd order polynomial to calculate barrier properties. The current was converted into current density by dividing the current with junction area.

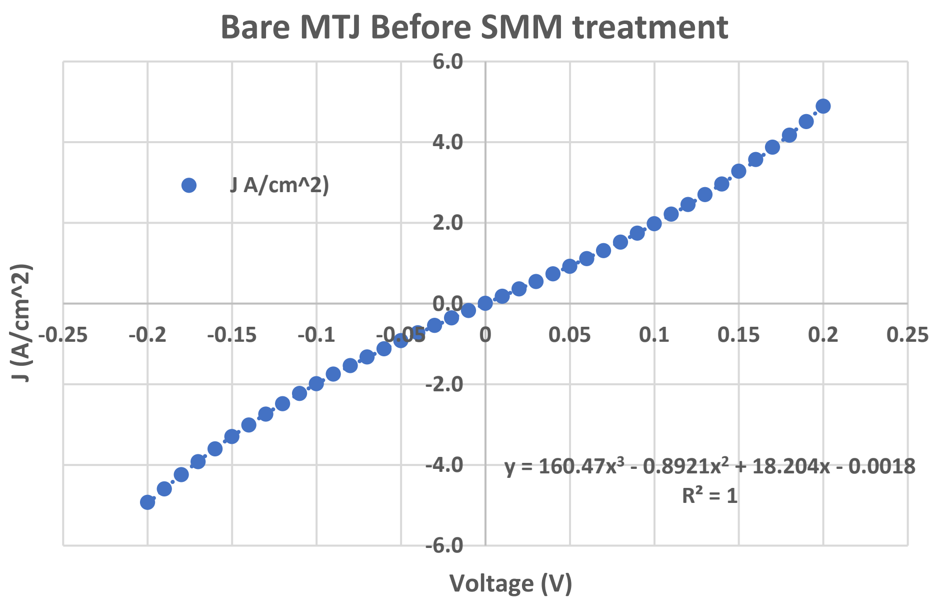

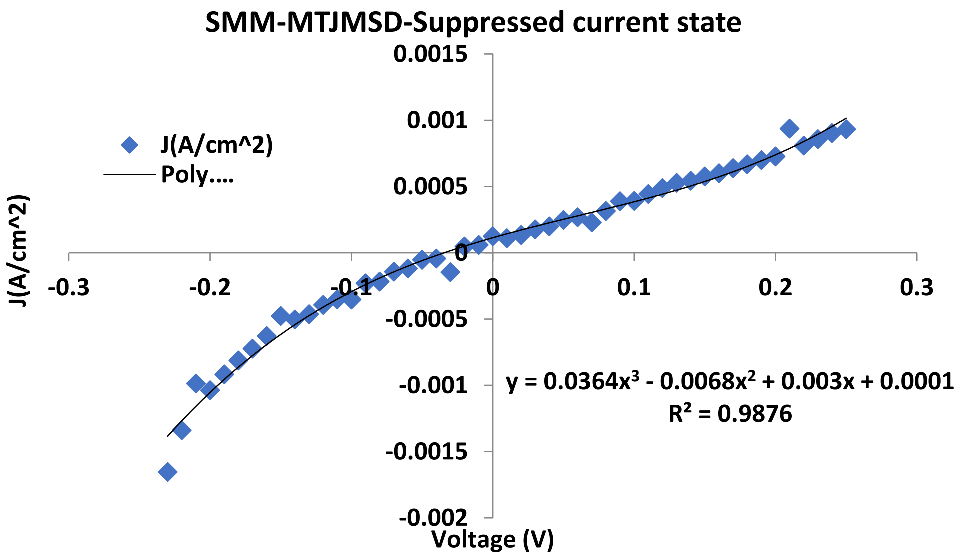

The equations for the Brinkman model are the following.

$\frac{G(V)}{G(0)} = 1 - \left(\frac{A\,\Delta\emptyset}{16\,\emptyset^{3/2}}\right) eV + \left(\frac{9\,A^2}{128\,\emptyset}\right) eV^2$ [Eq.1]

$G(0) = (3.16x10^{10}\emptyset/d)exp(-1.025d\sqrt{\emptyset})$ $[Eq.2]$

$A = 4(2\pi d/3h)\sqrt{(2m)}$ [Eq.3]

Where, G(V) is conductance at different bias V. The average of barrier heights of at the two interfaces is ø. The difference between two barrier heights at the two metal/insulator interfaces is Δø. The thickness of tunneling barrier is *d*. The G(0) and A parameters use in Eq.1 are described in Eq. 2 and Eq.3, respectively. The constant m and h are the mass of electron and plank's constant, respectively. The various forms of Binkman model is discussed elsewhere[11, 13]. We calculated barrier height ø and barrier thickness *d* of bare MTJ and SMM-MTJMSD in the high and suppressed current states and summarized in Table 1. According to the modeling data, the barrier thickness of bare MTJ was ~2.4 nm. This data is very close to our experimentally measured thickness of AlOx tunneling barrier that was ~2.1 nm [12]. Also, the barrier height of the AlOx tunneling barrier was ~0.6 eV and agreed with the ~0.7 eV barrier height estimated in our previous work by a different tunneling model[14]. Hence, our modeling appears to present a realistic view of the transport mediums utilized in the MTJ. As discussed, after SMM attachment, an MTJ became SMM-MTJMSD and assume a rather stable high current state.

Table 1: Calculated barrier height and thickness of MTJ before and after interacting with SMMs.

	Barrier thickness (nm)	Barrier height (eV)
Bare MTJ	2.42±0.09	0.56±0.06
In high current	1.31±0.44	2.06±1.24
In suppressed current	2.90±0.83	1.01±0.59

In the high current state SMM-MTJMSD's barrier thickness was ~1.3 nm, suggesting that transport is dominated via the additional SMM channels, not via the MTJ's AlOx tunneling barrier that was ~ 2 nm thick. Fig. 1c shows that SMM's core is connected to the Ni ferromagnetic electrode by the insulating ~1 nm long alkane molecule. To facilitate high current state, presumably, SMM foster transport in three stages (a) tunneling of electrons from Ni ferromagnet to SMM's core, (b) fast transport within SMM's core, and (c) tunneling of an electron from SMM to the opposite or second ferromagnetic electrode. Among three steps, tunneling via alkane tether is the slowest and dominates the overall molecular transport. This transport model is in close agreement with our previous MTJMSD studies utilizing another type of paramagnetic molecule [1, 12, 14]. However, the barrier height calculation in the higher current state showed a considerable variation and was calculated to be 2.06±1.24 eV. Fig. 2c showed that SMM-MTJMSD current kept changing. As also discussed in Fig. 4a, the SMM-MTJMSD in the high current state was sensitive towards magnetization. Experimental data in Fig. 2c and Fig.4a, suggests that SMM substantially modified the magnetic properties of the Ni ferromagnet. We have discussed molecule induced changes on the magnetic properties of the ferromagnets [15-17]. Interestingly, SMM-MTJMSD response in the suppressed current state was significantly different from bare MTJ and SMM-MTJMSD's high current state (Table-1). In the suppressed current state barrier thickness was 2.9±0.8 nm. It means that

SMM-MTJMSD's effective barrier thickness is more than the physical thickness of the AlOx tunneling barrier. This result is only possible when a part of Ni electrodes around interfacial regions got impacted by the SMM induced exchange coupling. We previously observed that a paramagnetic molecule was able to impact the complete junction area by establishing strong magnetic coupling with the ferromagnetic electrodes [15]. Affected magnetic electrode area, in the vicinity of AlOx tunneling barrier, appear to exhibit higher resistivity leading to increased barrier thickness in the suppressed current state[18]. However, in the present case of SMM-MTJMSD suppressed current state is not stable. The tunneling model based transport analysis has limitations and one should consider the impact of defects within the tunnel barriers, interfaces, and fitting quality [19-21]. The rationale for utilizing the tunneling model in the present paper is that our MTJ testbed is the result of previous optimization efforts[22]. We previously reported two steps ~ 2nm AlOx fabrication process[22]. This approach produced results that are congruent with the physical dimensions of the AlOx tunnel barrier and molecular channels. By no means, we claim the application of the tunneling model for molecular transport analysis to be ideal. However, this method has been producing results that have helped us understand the molecular transport on multiple occasions.[1, 18, 23]. It is noteworthy that options for analyzing molecular transport, especially with ferromagnetic electrodes, are extremely limited in scope. The main challenge is due to many assumptions required to simulate the MTJMSD (paramagnetic molecule connected between two large ferromagnetic electrodes). The first principle quantum mechanical approaches are unable to accurately account for large ferromagnetic electrode size, exchange interactions, anisotropies, and complex atomic configurations of SMM channels. Investigation of the refined atomic-level mechanism behind SMM based transport is required to investigate how the nature of the interaction between SMM and ferromagnets, present along the edges of an MTJ(Fig.1d), can produce a high current state and transient suppressed current state.

Monte Carlo Simulations

In order to understand the mechanism behind SMM molecule induced strong coupling we have conducted MC simulations on an analogous MTJMSD system designed in the Ising model framework (Fig.S6). To represent the molecules on the edges, (Fig. S6), a plane containing atoms along the sides and with empty interior was introduced between the two ferromagnetic (FM) electrodes; FM electrodes are represented by the Ising model. The inter-FM electrode magnetic coupling is only occurring via the molecules (Fig. S6). However, inter- FM electrode coupling via the empty space is considered to be zero. Using this MC model (Fig. S6) we performed MC simulations by varying molecular coupling strength with the top FM (J_{mT}) and bottom FM (J_{mB}) electrodes, kT and MTJMSD dimensions. To vary the dimension of a MTJMSD we varied the Height (H), width

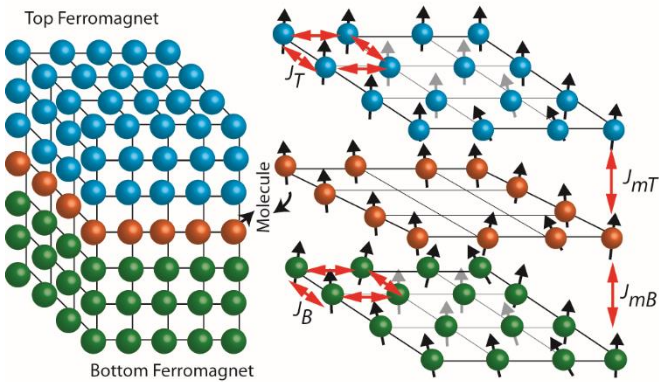

Figure S6. (Left) Ising Model of MJMSD. (Right) top ferromagnet and bottom ferromagnet interacting with paramagnetic SMM molecules present at the edges. Interaction among atoms and molecules is shown by the Exchange terms.

(W), and Length (L) and overall device dimension is represented by H x W x L (Fig. S6a). Molecular plane is inserted along the (H-1)/2th plane, i.e. the center plane along the H axis of a MTJMSD (Fig. 1j). To achieve the equilibrium state of a MTJMSD under the influence of molecule induced coupling we minimized the system energy as mentioned in eq. 1.

$$E = -J_T(\sum_{i\in T} \vec{S_i}\vec{S}_{i+1}) - J_B(\sum_{i\in B} \vec{S_i}\vec{S}_{i+1}) - J_{mT}(\sum_{i\in T, i+1\in mol} \vec{S_i}\,\vec{S}_{i+1}) - J_{mB}(\sum_{i-1\in mol, i\in B} \vec{S}_{i-1}\,\vec{S_i})$$
(Eq.1)

Where S represents the spin of individual atoms of FM electrodes and molecule in the form of a 3D vectors. In the eq. 1, J_T, and J_B, are the Heisenberg exchange coupling strengths for the FM electrodes on the top and bottom FM electrodes (Fig. S6b). Our MC studies utilized a continuous model1 which allowed spin vectors to settle in any direction according to the equilibrium energy governed by Eq. 1. For all MC simulations, the boundary condition was selected in such a way that the spin of atoms beyond boundary atom of the MTJMSD model (Fig. 1j) was zero.1 After choosing appropriate values for the Heisenberg exchange coupling coefficients, kT, and random spin states, a Markov process was set up to generate a new state. Under the Metropolis algorithm, the spin vector direction of a randomly selected site was changed to produce a new state; energy for the new and old configuration was calculated using eq.1. New states were accepted if the difference between the final and new energy (ΔE) was

$\Delta E<0$ *or* $\exp(-\Delta E/kT)\geq r$.

Where r is a uniformly distributed random variable whose magnitude range from 0 to 1. To achieve a stable low energy state, every MC simulation was run 10 to 100 million steps, depending upon MTJMSD dimensions. After this MC simulations, further runs were performed to generate an average magnitude of observables; two subsequent recordings for any observables were collected at the time interval comparable to autocorrelation time 1. The units of total energy E and exchange coupling parameters is the same as of kT. To keep discussion generic, the exchange coupling parameters and kT are referred to as the unitless parameters throughout this study. The overall magnetic moment of the MTJMSD is the sum of the magnetic moment of the two FM electrodes and the magnetic moment of the molecules.

We studied the magnetization (M) as a function of exchange coupling strength between SMM and top and bottom ferromagnets, JmT and JmB, respectively. For this study, we utilized 7x10x10 system size where each ferromagnetic electrode was of 3x10x10 dimensions. Molecules were placed in the middle, according to the schematic shown in (Fig. S6a). When molecules formed antiferromagnetic coupling with one ferromagnetic electrode and ferromagnetic coupling with another electrode, i.e., JmT and JmB possessed opposite sign, the MTJMSD magnetization decreased to zero. However, for this situation, at least one molecule-ferromagnet (JmT or JmB)coupling strength should be around 0.5 kTc [15].

However, in the event when the top and bottom ferromagnet electrodes formed strong ferromagnetic coupling or antiferromagnetic coupling with the molecules, then both ferromagnets aligned in the same direction. The magnetization of several MTJMSD with different electrode dimensions is shown in Figure S7b.

Hence, if molecule coupling with the two ferromagnetic electrodes switch signs, then overall MTJMSD's magnetization may switch drastically. When two ferromagnets are forced by molecular coupling to be

antiparallel, then current flow between ferromagnets is expected to be zero. Conversely, if SMM forced the ferromagnetic electrodes to be parallel to each other, the current flow between ferromagnets Will be the highest. High to low current transition on MTJMSD is also posible when the molecular coupling strength is of the same order as of the available termal energy. Hence, thermal energy plays a critical role in defining the effect of molecule induced exchange coupling impact. In the event when the thermal energy is comparable to the molecule induced antiferromagnetic coupling strength, overall MTJMSD magnetization may shuttle back and forth between the high and low magnetization leading to the switching between high and low current states as observed in the SMM based MTJMSD.

Follwoing two charts are for the larger MCS ising model of 19x10x10. It follows the same tren as discussed in the main manuscript.

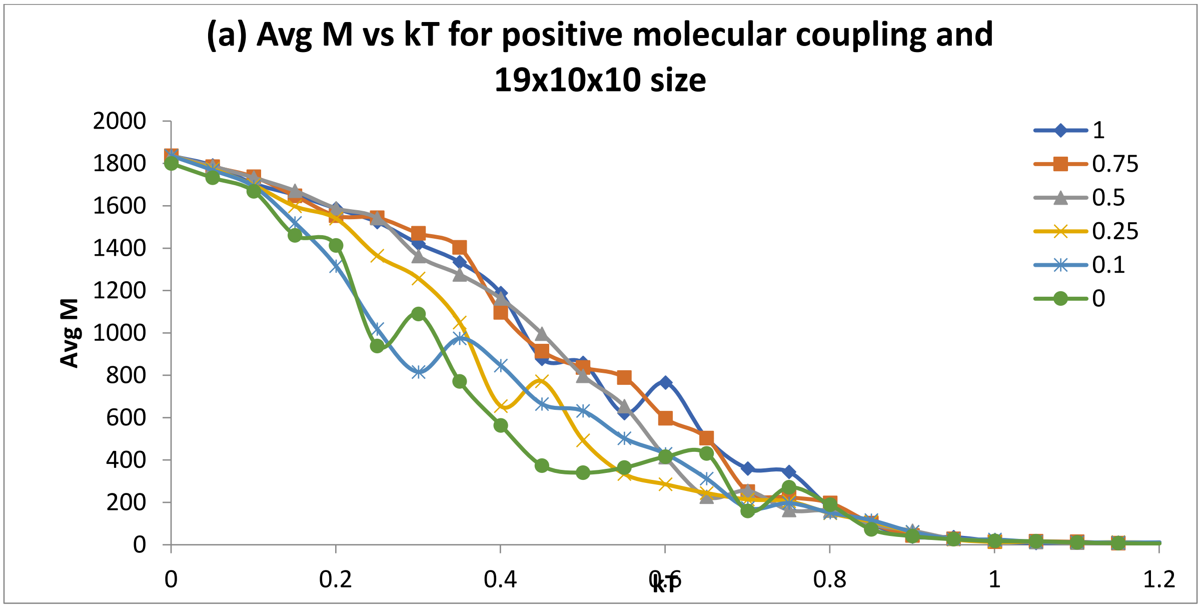

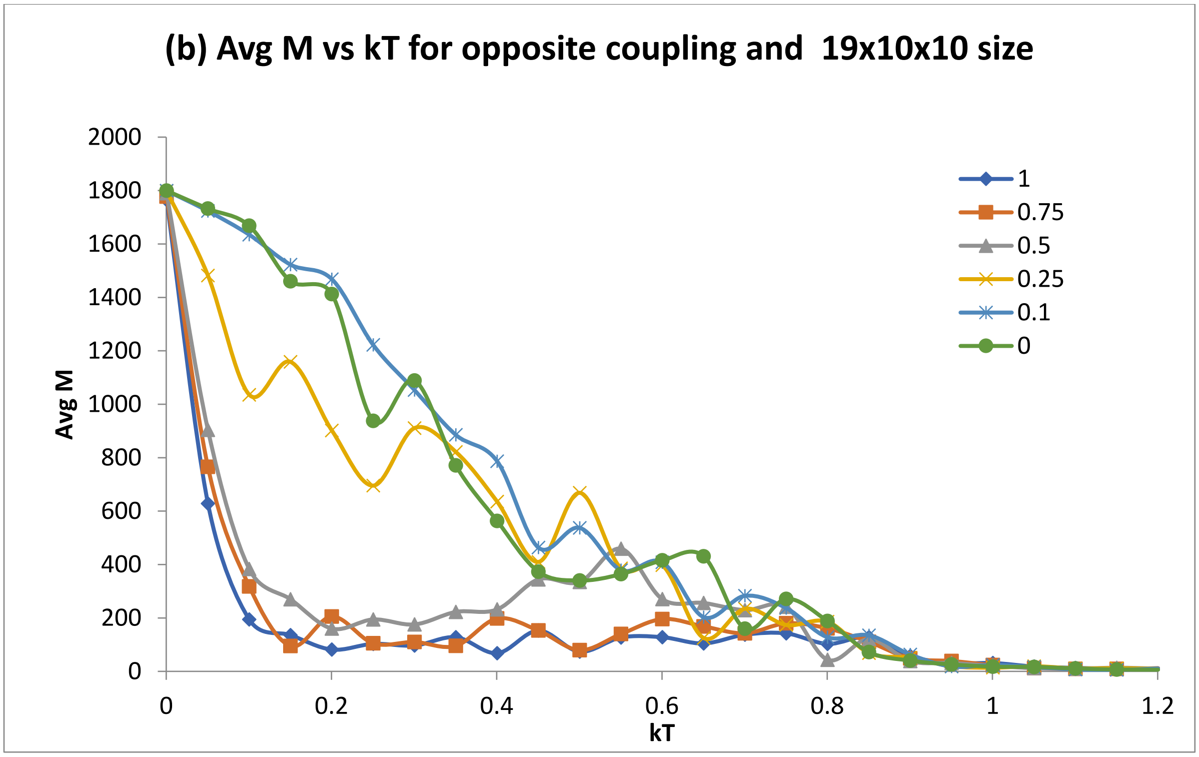

Fig. S7: Magnetization versus thermal energy (kT) graph for 3D Ising model (19x10x10) of SMM based MTJMSD when JmT and JmB are of same magnitude with (a) same sign and (b) opposite sign.

1. P. Tyagi, D. F. Li, S. M. Holmes and B. J. Hinds, J. Am. Chem. Soc. **129** (16), 4929-4938 (2007).
2. P. Tyagi, E. Friebe and C. Baker, J.Nanoparticle Res. **17** (11), 452 (2015).
3. C. Rojas-Dotti and J. Martínez-Lillo, RSC Advances **7** (77), 48841-48847 (2017).
4. A.-R. Tomsa, J. Martínez-Lillo, Y. Li, L.-M. Chamoreau, K. Boubekeur, F. Farias, M. A. Novak, E. Cremades, E. Ruiz and A. Proust, Chem. Comm. **46** (28), 5106-5108 (2010).
5. G.-Y. An, A.-L. Cui and H.-Z. Kou, Inorganic Chemistry Communications **14** (9), 1475-1478 (2011).
6. C. Rojas-Dotti, N. Moliner, F. Lloret and J. Martínez-Lillo, Crystals **9** (1), 23 (2019).
7. J. Martínez-Lillo, A.-R. Tomsa, Y. Li, L.-M. Chamoreau, E. Cremades, E. Ruiz, A.-L. Barra, A. Proust, M. Verdaguer and P. Gouzerh, Dalton Transactions **41** (44), 13668-13681 (2012).
8. J. Martínez-Lillo, N. Dolan and E. K. Brechin, Dalton Transactions **42** (36), 12824-12827 (2013).
9. J. Martínez-Lillo, N. Dolan and E. K. Brechin, Dalton Transactions **43** (11), 4408-4414 (2014).
10. J. Martínez-Lillo, J. Cano, W. Wernsdorfer and E. K. Brechin, Chemistry–A European Journal **21** (24), 8790-8798 (2015).
11. W. F. Brinkman, R. C. Dynes and J. M. Rowell, J. Appl. Phys. **41** (5), 1915 (1970).
12. P. Tyagi, J. Nanoparticle Res. **14** (10), 1195 (2012).
13. X. Hao, J. S. Moodera and R. Meservey, Physical Review B **42** (13), 8235-8243 (1990).
14. P. Tyagi and E. Friebe, J. Mag. Mag. Mat. **453**, 186-192 (2018).

15. P. Tyagi, C. Baker and C. D'Angelo, Nanotechnology **26**, 305602 (2015).
16. M. Cinchetti, V. A. Dediu and L. E. Hueso, Nat Mater **16** (5), 507-515 (2017).
17. S. Sanvito, Nat. Phys. **6** (8), 562-564 (2010).
18. P. Tyagi, C. Riso and E. Friebe, Organic Electronics **64**, 188-194 (2019).
19. J. J. Åkerman, R. Escudero, C. Leighton, S. Kim, D. Rabson, R. W. Dave, J. Slaughter and I. K. Schuller, J.Mag. Mag. Mat. **240** (1-3), 86-91 (2002).
20. C. W. Miller, Z.-P. Li, J. Åkerman and I. K. Schuller, Applied physics letters **90** (4), 043513 (2007).
21. C. W. Miller and D. D. Belyea, Journal of Applied Physics **105** (9), 094505 (2009).
22. P. Tyagi and B. J. Hinds, J. Vac. Sci. Technol. B **28** (5), 517-521 (2010).
23. P. Tyagi and C. Riso, Nanotechnology **30** (49), 495401 (2019).
